# Pathogenesis of Type 1 Diabetes Mellitus and Rodent Experimental Models

**Published:** 2018

**Authors:** I. G. Gvazava, O. S. Rogovaya, M. A. Borisov, E. A. Vorotelyak, A. V. Vasiliev

**Affiliations:** Koltsov Institute of Developmental Biology, Russian Academy of Sciences, Vavilova Str. 26, Moscow, 119334, Russia; Pirogov Russian National Research Medical University, Ministry of Health of the Russian Federation, Ostrovitianov Str. 1–9, Moscow, 117997, Russia; Lomonosov Moscow State University, Faculty of Biology, Leninskiye Gory 1–12, Moscow, 119234 , Russia

**Keywords:** beta-cells, diabetes, experimental laboratory animals, hyperglycemia, Langerhans islets, pathogenesis, streptozotocin

## Abstract

The global prevalence of diabetes mellitus and its severe complications is on the rise. The study of the pathogenesis of the onset and the progression of complications related to the disease, as well as the search for new therapeutic agents and methods of treatment, remains relevant. Experimental models are extremely important in the study of diabetes. This survey contains a synthesis of the most commonly used experimental animal models described in scientific literature. The mechanisms of the streptozotocin model are also analyzed and discussed, as it is considered as the most adequate and easily reproducible diabetes model. A review of the significant advantages and disadvantages of the described models has also been conducted.

## INTRODUCTION

Diabetes mellitus (DM) is currently one of the leading pathologies among the so-called diseases of civilization in terms of its high rates of incidence, disability, and mortality. According to the most recent data, there were 382 million people suffering from diabetes in the world in 2013, and the number of such patients is estimated to reach 592 million by 2035; i.e. an increase of 55% [1]. DM is a chronic condition characterized by a relative or absolute lack of insulin, which leads to hyperglycemia. Chronic hyperglycemia promotes the development of various complications, such as neuropathy, nephropathy, and retinopathy, and it also increases the risk of cardiovascular diseases. According to current classification, there are two main types of DM with numerous clinical, immunological, and genetic differences. Predisposition to DM is related to several groups of genes. It should be noted that the disease’s development is associated with certain alleles of the genes of the major histocompatibility complex (MHC) class II system. Aside from genetic factors, the genesis of diabetes mellitus involves environmental factors; thus the reference to this pathology as a multifactorial disease [2].

T1DM is an autoimmune disease associated with the destruction of the insulin-producing β-cells of the pancreas. T1DM is most often diagnosed amongst children and young people, and the production of endogenous insulin in patients is significantly down by the time of the diagnosis; therefore, regular insulin injections and continuous monitoring of blood glucose are necessary to reduce the risk of hyperglycemia. The most popular theory of T1DM pathogenesis was proposed by G.S. Eisenbarth [3]. According to this theory, T1DM develops in genetically predisposed individuals. Autoimmune processes in T1DM are triggered by environmental factors. The initial stage of T1DM – death of islet cells – is asymptomatic but can be detected by autoantibody tests. The clinical signs appear only at the latest stages, when most β-cells are dead, and absolute insulin deficiency develops [3, 4]. Initially, the genetics of T1DM were considered to be relatively simple. The presence of certain alleles of the HLA system genes was believed to lead to an almost complete dominance of the disease [5].

To date, there are more than 20 loci and 100 candidate genes that affect, to varying degrees, the development of T1DM [6]. However, the wide prevalence of this disease and the absence of significant correlations with genetic abnormalities suggest that people genetically not predisposed to the disease can also develop T1DM. For example, 85–90% of T1DM cases have been shown to occur in families without a primary history of T1DM in first-line relatives. V. Hyttinen and co-authors believe that the genetic predisposition amounts to about 30% [7].

The modern theory of T1DM pathogenesis proposed by M.A. Atkinson and G.S. Eisenbarth suggests that the disease’s development is facilitated or impeded by interactions among genes, rather than by a genetic predisposition [8]. In addition, these genes are believed to affect susceptibility and resistance to T1DM not only in the period preceding the induction of an autoimmune reaction, but also during the entire period preceding the disease.

T1DM symptoms are believed to manifest themselves usually when 90–95% of β-cells die [4]. However, there are many variations in this regard. In addition, the phenomenon of β-cell loss is not yet completely understood. The severity of this phenomenon is supposed to vary significantly depending on the type of insulitis, extent of β-cell death, and the β-cell ability to regenerate [9].

At present, there is no clear understanding of the mechanism of the autoimmune reaction that precedes the destruction of β-cells, in particular β-cell response to autoimmune antibodies.

According to modern concepts of T1DM pathogenesis, β-cells can die as a result of various pathological processes. One of these is the destruction or necrosis of β-cells, and another is apoptosis or genetically programmed cell death [10]. β-Cells undergo necrosis in the presence of an excessive amount of free radicals (oxygen radicals or nitric oxide) or under the action of pro-inflammatory cytokines [3, 11].

In recent years, the processes of necrosis and apoptosis have been demonstrated not to antagonize each other. Cytokines play an important role in the cell death process. Cytokines, such as IFN and IL-2, are considered triggers of insulitis, which are capable of activating a mechanism of signaling leading to the death of pancreatic β-cells [11].

Like all endocrine disorders, DM is a rather complex disease that involves various body systems. Despite the tremendous progress achieved in molecular genetics research, the issues of prevention and pathogenetic treatment of diabetes are yet to be developed to an adequate level. The main tool used in pathophysiology today is research conducted on experimental models; in this case, the choice of a model and its etiological and pathogenetic conformity to a human disease underlies not only the success of any theoretical study, but also the development of prevention and treatment modalities. Experimental models of DM provide valuable information for understanding the mechanism that underlies the antidiabetic action of various agents, which is necessary for their targeted use. To date, a variety of experimental DM models have been developed [12-16]. Objective assessment of the advantages and disadvantages of each model, in accordance with the target goal, is important to avoid erroneous results.

For more than 50 years, the only model of experimental diabetes mellitus has been diabetes induced by removal of the pancreas. The quantity of preserved pancreatic tissue is of paramount importance for the development of diabetic impairments in the postoperative period. Depending on this factor, diabetes can develop between a period of several hours (complete removal) and 9 months (removal of 80% of the organ). Subtotal pancreatectomy is often used to model chronic diabetes with a prolonged high blood glucose level. The main cause behind diabetes in this case is insulin deficiency: i.e., absolute insulin insufficiency. The use of this model at the first stage of experimental diabetology development enabled researchers to understand many aspects of the mechanisms of insulin action, the metabolic changes related to insulin deficiency, and the pathogenesis of diabetes-associated disorders. However, a number of the causes that complicate the use of operative removal of the pancreas have stimulated a search for new models. The emergence of non-operative models of DM sharply reduced the use of the previous method. In recent years, that method has been used in some cases to study the mechanism of action of natural compounds on insulin resistance and insulin secretion in various animals: rats, guinea pigs, and dogs. The effects of glucose uptake in various tissues upon removal of 90% of the pancreas and the significant hypoinsulinemia associated with subtotal resection of the organ, followed by additional resection, were studied [17, 18].

This review analyzes existing experimental models in an effort to identify the most adequate and widely used animal model of T1DM.

The main feature of type 1 diabetes mellitus is the autoimmune destruction of pancreatic β-cells, which leads to insufficient insulin production. Insufficient insulin production in animal models is caused by the action of many different mechanisms, ranging from chemical ablation of β-cells to the spontaneous development of autoimmune diabetes.

Genetic and non-genetic experimental models are used depending on the task at hand. Over recent years, the progress achieved in genetic engineering has resulted in the generation of many animals with genetically determined development of diabetes mellitus.

## SPONTANEOUS AUTOIMMUNE MODELS OF TYPE 1 DIABETES MELLITUS

In 1974, the so-called Non-Obese Diabetic (*NOD*) mouse strain was generated in Japan. These mice, along with other rodents such as *AKITA *mice, biobreeding (*BB*) rats, *LEW*.*1AR1 *rats, etc., are characterized by the ability to spontaneously develop autoimmune diabetes [16, 19, 20]. The spontaneous development of diabetes is likely associated with a genetic mutation affecting the selection of T-lymphocytes and leading to the impairment of the mechanisms of autotolerance control. *NOD *mice whose immunological characteristics are similar to those of insulin-dependent T1DM in humans have been routinely used as models of spontaneous autoimmune type 1 diabetes mellitus for the last 25 years [21-26]. These mice develop insulitis 3–4 weeks after birth. At this pre-diabetic stage, pancreatic islets are primarily infiltrated with CD4+ and CD8+ lymphocytes [27]. Insulitis causes the destruction of β-cells, but the pancreas of these animals produces up to 90% of its insulin until week 10–14, and the animals can develop diabetes up to the age of 30 weeks. In *NOD *mice, diabetes is more common among females (60–90%), while 10–30% of males develop the disease in most colonies [28]. *NOD *mice are characterized by the typical clinical symptoms of diabetes (hyperglycemia, glycosuria, polydipsia, and polyuria), but they do not develop ketoacidosis. If not treated with endogenous insulin, the animals die due to dehydration, not ketoacidosis, 2–4 weeks after the disease’s onset [23, 29]. In *NOD* mice, many genes are associated with a predisposition to T1DM and MHC alleles play an important role, as in humans, in this process. However, MHC class II alleles providing resistance or susceptibility to the disease in* NOD *mice have a structure that is different from that of human MHC class II alleles [27, 30, 31].

*NOD *mice are useful models in studying the genetics and mechanism of T1DM. These mice are potentially suitable for testing drugs that modulate the autoimmune response [23]. The advantages of *NOD *mice include the possibility of blocking cytokines by specific antisera and studying changes in the development and course of the disease [11, 32, 33]. It is this method that has been used to collect substantial data on the role of individual cytokines (interleukins, tumor necrosis factor, interferon γ) in the pathogenesis of autoimmune insulitis in diabetes [34]. However, it should be noted that, despite the high sensitivity of *NOD *mice to streptozotocin (STZ), β-cell death in them occurs in the absence of poly(ADP-ribose) polymerase (PARP) activation [35]. This fact can significantly affect the integrity of studies of β-cell sensitivity to diabetogenic factors in these animals [25, 26].

The initial optimism that accompanied the identification of a method for preventing T1DM by using animal models led to both discoveries and disappointments in the use of similar methods in humans. More than 192 methods that can be used to prevent T1DM in* NOD *mice have been reported [36-38]. Prevention of diabetes is relatively simple in mice, but it is extremely complicated in humans [5]. One of the causes may be that greater importance is attached to the similarity of T1DM in *NOD *mice and humans than to the differences [39]. In fact, diabetes in both mice and humans has a polygenic etiology characterized by impaired regulation of the immune response and the ability for remission after bone marrow transplantation. Differences in the action of maternal autoantibodies in mice and humans were revealed. In addition, there are differences in the incidence rate and gender. In *NOD *mice, the insulitis course is mild and benign [19]. Finally, there are significant differences in the functioning of immune systems in mice and humans [40].

Another commonly used model of autoimmune diabetes is *BB *rats generated from a colony of outbred Wistar rats in Canada (BioBreeding Laboratories) in the 1970s. Usually, after puberty, 90% of *BB *rats (males and females aged 8–16 weeks) develop spontaneous diabetes with a rather severe phenotype and the need for insulin therapy [41]. The animals have insulitis with the presence of T cells, B cells, macrophages, and NK cells, but with a sharply decreased number of CD4+ T cells and almost complete absence of CD8+ T cells. T cell lymphopenia characteristic of these animals is not typical of T1DM in humans and *NOD *mice and is considered as the model’s drawback. It should be noted that insulitis in *BB *rats is not preceded by peri-insulitis [42]. However, *BB *rats are used as a small animal model for the induction of tolerance after islet transplantation [41], as well as for the investigation of diabetic neuropathy.

## GENETICALLY INDUCED INSULIN-DEPENDENT DIABETES

*AKITA *mice were generated in Japan from C57BL/6NSIc mice with a spontaneous mutation in the *ins 2 *gene, which prevents correct pro-insulin processing and leads to endoplasmic reticulum stress (ER stress). Starting at the age of 3–4 weeks, mice with this mutation develop insulin-dependent diabetes that is characterized by hyperglycemia, hypoinsulinemia, polyuria, and polydipsia. The absence of β-cell mass in this model makes it an alternative to the STZ-induced model used in transplantation studies [22]. *AKITA *mice are also used as a model of T1DM in studies of macrovascular diseases [43] and neuropathies [44]. This model has been widely used to investigate potential ER-stress suppressors in pancreatic islet cells: therefore, *AKITA* mice can be used to study certain pathologies associated with T2DM [45].

However, the results obtained in rodents cannot be used in clinical medicine because there are both specific differences in the immune system of rodents and humans and the species-specific features of pancreatic Langerhans islets. Human and mouse islets that are intended for use as targets for autoimmune attack differ in many aspects, including the architecture and composition of the cells, proliferative activity, susceptibility to injuries, and ability to form islet amyloid, as well as in the expression of heat shock proteins, islet transcription factors, antioxidant enzymes, and the main glucose transporter (GLUT-1 or GLUT-2). For example, the inner β-cell mass in rodents is surrounded not by β-endocrine cells, whereas endocrine islet cells in humans are more mixed. In addition, unlike rodent β-cells capable of restoring or regenerating in response to some stimuli (insulin resistance, β-cell ablation, and partial pancreatectomy), the proliferative potential of human β-cells is either very small or absent [46].

The differences in the immune system of rodents and humans are primarily associated with the major histocompatibility complex (MHC). Transplantation of human immune cells and tissues onto immunodeficient mice produces the promising mouse models used to study natural human immune responses. There have been attempts to improve experimental models of diabetes mellitus by using humanized transgenic mice expressing human MHC class II molecules that predispose to diabetes. There have been new strains of immunodeficient mice suitable for the survival of grafted human functional tissues, including hematopoietic stem cells, mature lymphocytes, and pancreatic islets. For example, *NOD*-*SCID *mice were used to develop unique strains of *NSG *mice with a targeted mutation of the *IL2rynull *receptor common γ chain. *NSG *mice are considered perfect for studying the functions of the human immune system *in vivo *and determining the action mechanisms of drugs in T1DM [47-50].

Models based on immunodeficient mice have a number of disadvantages. First, they have natural killer (NK) cells, and human pancreatic islets are very sensitive to NK cells. Second, they do not enable engraftment of the functional human immune system [37, 39]. Deficiency of the *IL2rynull *receptor common γ chain completely blocks NK cells, causing additional defects of innate immunity. *NSG *mice are completely devoid of NK cells. *NSG *mice are a convenient model for studying the functions of transplanted islets of the human pancreas in the absence of the potential toxic effects of glucose, despite the fact that the euglycemia (120–160 mg/dL) in these animals is characterized by a higher blood glucose level in contrast to that in humans (80–100 mg/dL). Normoglycemic *NSG *mice are available in an unlimited amount; cells transplanted to them are not exposed to high glucose levels; fewer cells are required for an analysis of the function than for the regulation of hyperglycemia in recipients with diabetes [47-50]. Therefore, *NSG *mice have been shown to be readily available; they enable optional induction of hyperglycemia and restoration of normoglycemia by grafting Langerhans islets and suspensions of human and mouse pancreas cells; most important, these mice can be grafted with a functioning human immune system. However, when these mice are exposed to STZ, they, despite a number of the described advantages, also exhibit disadvantages: unstable induction of hyperglycemia, possibility of using endogenous mouse islets to restore normoglycemia, and STZ toxicity.

Genetic models of hyperglycemia were developed to induce hyperglycemia without the use of toxic compounds [47, 48]. These include the mouse models* NOD-Rag1^null^ Prf1^null^ Ins2^Akita^*, *NOD-Rag1^null^ IL-2ry^null ^Ins2^Akita^*, etc. The advantages of these models include: 1) spontaneous development of hyperglycemia without the use of toxic agents; 2) persistent and severe hyperglycemia; 3) no return to normoglycemia, due to endogenous mouse islets; 4) no need for exogenous insulin to prevent the development of metabolic decompensation and death. These models are able to support engraftment of the functional human immune system; therefore, they may be used to study alloimmunity and autoimmunity.

Despite the significant contribution of research on genetically modified animals to our understanding of the mechanism of diabetes pathogenesis, their role should not be overestimated. When using these models, acquired predisposition issues that play an important role in the development of type 1 diabetes mellitus may remain out of sight. T1DM is known to be strictly genetically determined in only 6–7% of cases, while in other cases the disease develops without significant hereditary predisposition [51]. The disease was found to develop not in all carriers of diabetes-associated alleles [52]. Therefore, experimental studies of the action mechanisms of unfavorable environmental factors seem promising. In this case, β-cell death mechanisms are largely versatile and independent of the acting factor, which enables an extrapolation of the results obtained in experimental models to humans [52].

## CHEMICALLY INDUCED TYPE 1 DIABETES MELLITUS

Chemically induced T1DM is associated with the destruction of a large number of endogenous β-cells, which leads to a reduced production of endogenous insulin, followed by the development of hyperglycemia and weight loss. Chemically induced diabetes in rodents and higher animals is a simple and relatively cheap model of this disease [53].

T1DM induced by chemical substances (STZ, alloxan, dithizone) is appropriate for an evaluation of drugs or therapeutic approaches that decrease the blood glucose level independently of β-cells; for example, for testing new insulin forms [54, 55]. This model is also appropriate for assessing the effectiveness of transplantation therapy that also reduces the blood glucose level [56, 57]. It is considered necessary to exclude spontaneous regeneration of β-cells in transplantation [58, 59] and also to perform a histological study of the endogenous pancreas for identifying insulin-positive cells and measuring the insulin level [59]. However, in the case of a chemically induced model of T1DM, the presence of β-cells has been shown not to necessarily correlate with their function [60].

One of the drawbacks of chemically induced diabetes is the potential toxicity of chemicals to other organs. It should be noted that administration of STZ and alloxan has been associated with changes in the expression level of P450 isoenzymes in the liver, kidneys, lungs, intestines, testes, and brain. This fact should be considered when testing drugs in animal models [61].

The STZ-induced model of T1DM is the most widely used at this moment. It has replaced the alloxan model [36, 40], the essential drawbacks of which are associated with the neurotoxicity and nephrotoxicity of alloxan and the lack of a clear dose-response relationship.

The natural antibiotic STZ is produced by *Streptomyces achromogenes *actinomycetes; this is N-acetylglucosamine (2-deoxy-2-(3-methyl-3-nitrosourea)-1- D-glucopyranose) that contains a nitrosourea moiety in lieu of acetate [36, 37]. STZ exhibits antibacterial and antitumor activities and is used in antitumor therapy. However, STZ has been found to cause the development of hypoglycemic conditions. STZ has been shown to be capable of inducing specific necrosis of β-cells in laboratory animals. The observed insulinemic syndrome has been called streptozotocin-induced diabetis, and STZ has been used to induce experimental T1DM. Let us consider this model in more detail.

## STREPTOZOTOCIN-INDUCED DIABETES MELLITUS

Currently, streptozotocin diabetes is induced in most laboratory animals: rats, mice, guinea pigs, rabbits, dogs, and monkeys. However, different animal species, even within the same family, often differ significantly in sensitivity to STZ. Investigation of interspecies and intergroup differences in resistance to STZ is one of the important tasks of experimental diabetology. It is believed that rodents, especially rats, are most sensitive to STZ, while humans and fish are maximally resistant to it [62]; in this case, human β-cells are much more resistant to STZ than the β-cells of other anthropoid primates [62]. This phenomenon is genetically determined and is associated with the expression of different types of glucose transporters on the cell membrane, the features of enzymatic glucose oxidation systems in mitochondria, and differences in the DNA repair system [63].

## INTRASPECIES DIFFERENCES

There are significant intergroup differences in the resistance to STZ within the same species. Inbred strains of rats and mice differ widely in their sensitivity to STZ [64, 65]. The diabetogenic action of STZ is enhanced by androgens and inhibited by estrogens, which leads to significant differences in the sensitivity to STZ in males and females [66]. An important role in sensitivity to diabetogenic factors is played not only by gender-related differences, but also by certain individual characteristics. For example, Wistar rats may be allocated into three groups of animals with differing resistance to diabetogenic factors, which manifests itself in the number of β-cells that die when exposed to STZ. This heterogeneity is related not to the breadth of the reaction norm but to the existence of isolated groups of animals with differing resistance. Animals of the first group are characterized by rapid development of hyperglycemia and significant destruction of pancreatic islets already at the initial stages of diabetes. The second group is characterized by a prolonged latent course of the pathological process when fasting euglycemia is associated with impaired glucose tolerance. The third group that is characterized by periodically occurring hyperglycemia falls in between the first two groups [63].

## STZ DOSES AND ADMINISTRATION PROCEDURES

Single administration of a high STZ dose leads to the development of hyperglycemia, and experimental models of laboratory rodents generated in this way may be useful in grafting and testing insulins. Multiple administrations of low STZ doses are also used to model T1DM. However, T1DM models based on the development of autoimmune insulitis have been developed only in a few strains of mice with a genetic predisposition [62, 67]. This method is not appropriate for the generation of an adequate model of human T1DM in other animal species [62]. In these cases, a single injection of a diabetogenic dose of STZ (which depends on the animal species) is desirable to use for the induction of a self-progressive pathological process with an autoimmune component [62].

Diabetogenic doses of STZ, like procedures of their administration, are different for different animal species. The sub-diabetogenic dose of STZ for rats is 25 mg/kg, with the optimal diabetogenic dose being about 50–75 mg/kg [62, 68, 69]. In most animals, this dose leads to diabetes manifestation with hyperglycemia, hypoinsulinemia, dyslipidemia, and significant destruction of pancreatic islets, in combination with their lymphoid infiltration. For other rodent species, diabetogenic doses are significantly higher and range from 100 to 200 mg/kg [62, 70]. Fish β-cells show high resistance to STZ that, even at high doses (350 mg/kg), causes only short-term impairment of insulin synthesis and secretion, without destruction of pancreatic islets. This phenomenon is associated not with accelerated degradation of STZ in the liver or kidneys, but with the peculiarities of β-cell metabolism in these animals. Because of the instability and short half-life of STZ, its intravenous administration is considered to be the most reliable. However, there are also other ways of administering the drug to induce experimental diabetes: the intraperitoneal method and direct infusion into the pancreatic vessels. STZ is stable only at low temperatures in an acidic medium, while under neutral and alkaline conditions, it rapidly (within a few minutes) degrades to inactive metabolites lacking diabetogenic effect [71]. For this reason, STZ, dissolved *ex tempore*, should be administered in citrate buffer at acidic pHs ~ 4.5 [66].

## PATTERNS OF EXPERIMENTAL T1DM DEVELOPMENT

The blood glucose concentration changes in response to a change in the plasma insulin concentration after STZ administration [72]. These changes occur in three phases. Unlike alloxan, STZ does not inhibit glucokinase. One hour after administration, the first hyperglycemic phase starts; it reaches a maximum after 2 h and lasts up to 4 h. The development of early hyperglycemia is believed to be caused by the suppression of insulin secretion due to the toxic effect of STZ on pancreatic β-cells [73]. Some authors associate it with an increased rate of hepatic glycogenolysis or consider it as secondary to the elevation in the free fatty acid content [13, 74]. Ultrastructural changes in the synthesis and energy apparatus of β-cells, which are accompanied by disruptions in the biosynthesis of proinsulin and insulin, were observed in the hyperglycemic phase [75]. After 4–8 h, the next hypoglycemic phase occurs, which lasts for several hours (up to a day) and is considered to be caused by the release of insulin from damaged β-cells. Loss of secretory granules develops in association with irreversible changes in subcellular organelles and nuclei. The final phase of the glycemic curve is characterized by persistent hyperglycemia and the development of permanent diabetes 24 h after STZ administration. Morphological and ultrastructural analyses indicate complete degranulation and disintegration of β-cells. Secondary hyperglycemia is considered as the result of absolute insulin deficiency.

According to other authors, hyperglycemia in experimental STZ-induced diabetes also develops in several consecutive stages but they are more prolonged: for example, the primary hyperglycemic reaction for 1–4 days; a period of euglycemia in the setting of impaired glucose tolerance (5–9th day); and a period of stable hyperglycemia, hyperphagia, and polyuria (10 days and more) [66]. Twelve hours after STZ administration, a primary hyperglycemic response develops; it is caused by the death of a significant portion of β-cells in pancreatic islets. The peak of hyperglycemia occurs on day 2 or 3, followed by a short period of euglycemia. This is associated with the potential ability of β-cells to enter mitosis under the influence of a high glucose concentration [5]. Activation of β-cell proliferation is believed to occur on the 3^rd^ day of diabetes [63]. An increase in the β-cell mass further leads to a rapid decrease in glycemia to physiological values (before the end of day 9) and corresponds to incomplete compensation of the pancreatic insulin apparatus function. Inadequacy of the compensatory response is manifested as impaired glucose tolerance. By the 10–14^th^ day, animals experience a repeated increase in the glycemia level [63]. Probably, an expanded autoimmune response to the neoantigens of pancreatic islets develops during this period, which leads to the death of most β-cells, fibrosis and sclerosis of the islets, and proliferation of alpha-cells. In preserved β-cells, glucose-induced insulin secretion is largely impaired. This is associated with several causes: a nonspecific response of β-cells to any damaging factors (including STZ) and the specific action of IL-1B and NO on glucose metabolism in mitochondria, which disrupts normal activation of β-cells [75, 76]. In addition, activated islet macrophages and T lymphocytes produce the neuropeptide γ (NPY) that inhibits insulin secretion [76].

## FACTORS UNDERLYING DIFFERENCES IN RESISTANCE TO STZ

The differences in sensitivity to STZ are largely caused by intracellular events occurring after the transfer of STZ through the cell membrane to the cytosol and before depletion of NAD^+^ stores [63]. According to published data, resistance to STZ is controlled by a number of factors.

1. Sensitivity to diabetogenic action depends primarily on the physiological properties of β-cells. This underlies the differences in the rate of inactivation and excretion of STZ [62, 71].

2. A different degree of expression of type 2 glucose transporters (GLUT-2) that are specific STZ carriers into the cytoplasm of β-cells. High resistance of human β-cells to STZ is caused by preferential expression of GLUT-1, not GLUT-2, on the β-cell surface [62].

3. Differences in the activity of oxidation systems; for example, a lower activity of glycolysis enzymes causes greater susceptibility to and toxicity of STZ in voles than in mice.

4. Intracellular accumulation of various STZ metabolites, some of which promote, like STZ, the generation of free radical products and mutations [77, 78].

5. Differing sensitivity of inbred mouse strains to STZ is caused by a difference in the activity of poly(ADP-ribose) polymerase (PARP) [77].

6. Expression of heat shock proteins that are potent factors of pancreatic β-cell resistance to the toxic effect of STZ. The level of their expression in both nonspecific (effect of STZ) inflammation and autoimmune inflammation is considered as one of the most important parameters controlling the viability of β-cells in insulitis [79, 80].

7. Differences in the activity of antioxidant systems, in particular the higher activity of glutathione peroxidase in mice, are one of the factors underlying the high resistance of mice to STZ [63].

8. Transgenic mouse strains expressing interferon γ in β-cells are significantly more resistant to the induction of diabetes than the initial strain [81]. This example demonstrates the role of intra-islet paracrine factors [81]. However, the molecular mechanisms of this phenomenon remain as yet not fully understood.

9. The cumulative effect of damaging factors is also considered as a cause of the differences in resistance to STZ. Adverse environmental factors (especially those acting during early ontogeny) that cause a stress response through a high level of glucocorticoids and changes in the neuroendocrine regulation of the pancreatic islet function can lead to endocrine imprinting associated with significant rearrangement of the intracellular systems of β-cell function regulation [63]. In adult animals that have undergone stress in the prenatal period, glucose-stimulated insulin secretion and glucose tolerance are impaired and the sensitivity of β-cells to the toxic effect of STZ is significantly increased [5].

The tropicity of STZ to β-cells is controlled by a glucose residue in the STZ molecule [82], which enables its selective binding to the GLUT-2 glucose transporter and transport to the cytoplasm [83]. Therefore, cell sensitivity to STZ depends on the expression of the GLUT-2 carriers that are expressed exclusively by pancreatic islet β-cells in most animals. This is also confirmed by the following observations: insulin-producing cells not expressing the glucose transporter are resistant to STZ; they become sensitive to the toxic effect of the drug only after the expression of GLUT-2 in the cell membrane [84]. In addition, other cells expressing this transporter, such as hepatocytes and epithelial cells of the renal tubules, are also exposed to the toxic action of STZ. Therefore, administration of STZ to animals leads to the development of not only diabetes, but also damage to the liver and kidneys [63].

## TOXIC EFFECT

STZ is capable of non-enzymatic release of free NO associated with the toxic effect of STZ [39, 85]. In this case, islet β-cells accumulate a large amount of STZ, which results in a high concentration of NO that, being in a liquid medium, rapidly transforms into peroxynitrate, which leads to the activation of free radical oxidation processes [39]. This leads to disruption of the cell membrane integrity, reduced efficiency of oxidative phosphorylation in mitochondria [37, 67], and point mutations in DNA, such as covalent modification of purine bases and the emergence of N-7-methylguanine, O-6-methylguanine, and 3-methyladenine. STZ and its metabolites are alkylating agents that methylate guanine and, to a lesser extent, adenine residues in DNA [86]. DNA damage leads to the activation of repair systems. The key enzyme involved in the repair of point mutations is PARP that replaces a defective base with a poly-ADP-ribose tail [59, 77]. The repair process requires NAD, which, given the huge number of NO- and STZ-induced mutations, leads to depletion of the cell NAD pool and cell death [71, 87, 88]. In this case, transgenic mice with PARP deficiency are resistant to diabetogenic factors. Recent studies have shown that, although STZ also methylates proteins, it is DNA methylation that is responsible for the death of β-cells [73]. The ability of STZ to cause energy deficiency in cells was shown to play the decisive role in its toxic effect towards β-cells [71, 87, 88].

PARP is the key factor involved in the death of β-cells [37, 62]. PARP is activated regardless of whether DNA damage is caused by chemical factors (STZ, alloxan), inflammatory factors (NO, cytokines, reactive oxygen species), or β-cytotropic viruses [62, 89].

On the other hand, the specific effect of NO on β-cells also includes activation of guanylate cyclase, an increase in the cGMP level, and inhibition of mitochondrial aconitase, which leads to impairment of aerobic glucose oxidation and, as a consequence, suppression of glucose-stimulated insulin secretion and synthesis [67, 90, 91]. In this case, inhibition of aconitase (that participates in the Krebs cycle) in the setting of PARP hyperactivation leads to complete depletion of intracellular NAD and ATP stores, which is the direct cause of β-cell necrosis. In the case of STZ-induced β-cell death, apoptosis processes are also blocked due to complete depletion of intracellular ATP and NAD stores [92, 93].

NO generation is responsible for both the initiation and development of diabetes caused both by viruses [89] and toxic substances [94] and by an autoimmune response. The alkylating agent methyl methanesulfonate, being the most toxic compound, is not a donor of NO; thereby proving that NO is unnecessary to the toxic effect of alkylating agents, including diabetogenic streptozotocin. NO and free nitroxide radicals can enhance STZ toxicity, but NO is certainly not a decisive factor of toxicity to β-cells [51]. However, the ability of STZ to cause ATP pool depletion and, therefore, energy deficit is important to the toxic effect on β-cells. The biological effects of STZ on the homeostasis of glucose and insulin are a result of damage to β-cells. On the one hand, glucose homeostasis disturbance (oxygen consumption and glucose oxidation) and inhibition of insulin biosynthesis and secretion are obvious. On the other hand, STZ has been found not to immediately and directly affect the transport of glucose or its phosphorylation by glucokinase [33, 95]. Inhibition of insulin biosynthesis and secretion is supposed to be initially caused by STZ-induced depletion of NAD^+^ [96].

Cytokines, such as IL-1, in the immunocompetent and endocrine cells of pancreatic islets have been shown to trigger the expression of the inducible nitric oxide synthase (iNOS) [85, 97] that produces significant amounts of the major biological mediator and, thus, causes β-cell death [39, 98].

Therefore, STZ activates the same pathogenetic mechanisms (suppression of glucose oxidation, DNA mutations, NAD depletion) as those activated by other poisons and viruses toxic to β-cells, and the key agents implementing these processes, regardless of the damaging factor, are NO and PARP. Thus, it should be concluded that the streptozotocin model of diabetes is etiologically and pathogenetically very close to human T1DM.

Despite the variety of experimental DM models reported to date, STZ-induced diabetes is the preferable one. The advantages of this model include relatively simple reproducibility, a highly selective effect, and induction of diabetes of varying severity and duration, which enables the modeling of both progressively developing β-cell dysfunction and impaired glucose tolerance with associated disorders. A number of the disadvantages of nongenetic STZ-induced diabetes models (scattering of glycemia level data, spontaneous normalization of insulin secretion function) can be eliminated by a judicious choice of the diabetogenic dose of the drug and adequate planning of the experiment.

Experimental DM models in laboratory rodents are undoubtedly a very useful tool for studying the pathophysiology and clinical aspects of the disease and are used as the first step in the investigation of new promising therapies. However, animal models, in general, and rodent T1DM models, in particular, are imperfect and exhibit certain drawbacks when their results are extrapolated to humans. Furthermore, the results obtained in rodents may sometimes prove misleading when studying the prevention of T1DM [64, 99]. To avoid compromised results, a degree of caution is necessary when choosing the model and drug dose for the induction of experimental diabetes. It is necessary to standardize the models and experiments specifically for studies of DM prevention, clearly interpret reliable results, and create a database after multiple iterations of the experiments.

The question of to which extent the results obtained in models may be extrapolated to humans is both the most important and most difficult one when laboratory animals are used [100, 101]. However, the question of how relevant a particular model is to the processes occurring in the human body remains open. Evaluation of the adequacy of experimental models includes a body of evidence that demonstrate that the results obtained in animals may be, to a certain degree, extrapolated to humans.

The data presented here do not reflect the entire spectrum of the T1DM models developed to date. The number of models is constantly growing, but they have not been sufficiently explored. In this case, it should be remembered that each experimental model simulates only certain aspects of the T1DM pathogenesis and does not completely match the development and course of the disease in humans. Therefore, there is ongoing research on the modification of existing models and development of new, more advanced models that most adequately reflect the changes typical of T1DM in humans.

It should be emphasized that adequate modeling of T1DM is a necessary basis for the preclinical testing of antidiabetic agents, and the use of various models enables to substantiate the extrapolation of experimental results to T1DM patients.

## Abbreviations

T1DM: type 1 diabetes mellitus
STZ: streptozotocin
β-cells: beta-cells
